# Antimicrobial resistance in the farm-to-plate continuum: more than a food safety issue

**DOI:** 10.2144/fsoa-2020-0189

**Published:** 2021-03-02

**Authors:** Luria L Founou, Raspail C Founou, Sabiha Y Essack

**Affiliations:** 1 Department of Food Safety & Environmental Microbiology, Centre of Expertise & Biological Diagnostic of Cameroon, Yaoundé, Cameroon; 2 Antimicrobial Research Unit, College of Health Sciences, University of KwaZulu-Natal, Durban, South Africa; 3 Bioinformatics & Applied Machine Learning Research Unit, EDEN Foundation, Yaoundé, Cameroon; 4 AMR Insights Ambassador Network; 5 Department of Biomedical Sciences, Higher Institute of Medical Technology, Yaoundé, Cameroon; 6 Department of Microbiology-Hematology-Immunology, Faculty of Medicine & Pharmaceutical Sciences, University of Dschang, Dschang, Cameroon

**Keywords:** animal health, antimicrobial resistance, climate change, foodborne illness, food safety, food security, human health, sustainable development, United Nations

## Abstract

Antimicrobial resistance (AMR) threatens to reverse the essential benefits of antibiotics, not only in humans, where decades of advancements in healthcare outcomes are endangered, but also in the food production industry. As the world moves toward Sustainable Development Goals, food safety is a critical element to improve and strengthen global health, and ensure sustainable development. Emergence of AMR in the food production industry represents a serious risk for exposed workers, their relatives and consumers. This perspective presents the challenge of AMR through the lens of food safety, by highlighting its multisectoral and multidimensional implications not only on the Sustainable Development Goals for food safety and public health but also on food security, animal health and welfare, the environment and climate, and socioeconomic development.

## Lay abstract

Antimicrobial resistance (AMR) occurs when microorganisms are unaffected by antimicrobials that previously killed/inhibited them and cured the infection. This paper focuses on AMR from a food safety perspective. AMR in the food chain is a risk to exposed workers and consumers both locally and globally. AMR in the food chain affects the achievement of Sustainable Development Goals related to poverty, hunger and promoting health and well-being. The consequences of AMR in the food chain thus extend beyond reduced productivity and food safety to reach food insecurity, becoming a drain on national and global economies as well as worsening climate change.

Generally, agriculture is the group of technical processes and works in natural fields including terrestrial and aquatic environments, which allow the production of food products. It encompasses several activities including crop production, livestock breeding, fishing and hunting, all of which are associated with the farm-to-plate continuum. The availability and use of antimicrobials, especially antibiotics, in the food production industry (*viz*., terrestrial and aquatic animal and crop production) is indispensable to maintain animal health and welfare, sustain productivity, contribute to food safety and security, and for the protection of livelihoods [1]. However, the increasing worldwide concerns related to antimicrobial resistance (AMR) threatens the reversal of essential benefits of antibiotics not only in humans, where decades of improvements in healthcare outcomes are endangered but also in the food production industry [1].

AMR is a general term that encompasses the resistance of bacteria, fungi, parasites, viruses to antimicrobials normally active against them, whereas antibiotic resistance (ABR) is a more specific concept of bacterial resistance to antibiotic substances [2]. For the purpose of this article, the commonly used term AMR is retained, albeit with the emphasis on ABR. As an ancient and natural evolutionary process occurring whenever antimicrobial substances including antibiotics are used [2], the rate of AMR emergence and spread has far outstripped progress in the development of new and effective antimicrobial drugs, leading the world toward a post-antibiotic era devoid of these life-saving substances [1]. Although antibiotic use in human health has initially been established as the most important risk factor for the emergence and spread of AMR, the concern has been exacerbated due to the extensive use of antimicrobial drugs in the food animal production [3]. It is now widely acknowledged that increased AMR in bacteria affecting humans and animals is also influenced by the extensive usage of antimicrobials in animal production for a variety of purposes, including therapeutic and nontherapeutic uses as metaphylactics, prophylactics and growth-promoters [1,4].

Emergence and spread of antibiotic-resistant bacteria (ARB) and antibiotic-resistant genes (ARGs) in the farm-to-plate continuum (*viz*., from the farm to the end consumer) via direct and indirect contact has health and socioeconomic repercussions globally. Direct contact that occurs through immediate exposure of humans with infected or colonized food animals and their biological substances, enhances the rapid and easy dissemination of ARB and ARGs from host-to-host. The human population may also be exposed indirectly to ARB and ARGs via contact with or consumption of contaminated food products [3]. This indirect transmission has been associated with several foodborne illness outbreaks globally and is often considered more dangerous as it indicates the transfer or spread of AMR at each step in the continuum [5,6]. Direct and indirect transmission of ARB and ARGs in the food chain increase the likelihood of their entrance and spread into communities and hospitals where substantial exchanges are possible, potentially jeopardizing healthcare systems [3]. Moreover, with the globalization of trade in animals and food products as well as international travels, there are no species, ecological nor geographical boundaries to contain AMR. Resistance emerging in one geographical location or bacterial species can easily spread or spill-over into several bacteria at each stage in the farm-to-plate continuum and similarly affect all countries, regardless of the income level [3]. AMR is thus a quintessential One Health issue requiring global solutions [7].

Determining the relative implications of AMR emergence and spread in food animal production is a significant challenge given the interconnectedness and interdependence of epidemiological pathways between animals, humans and the environment [8]. The United Nations (UN), the WHO, the United Nations Food and Agriculture Organization (FAO), the World Organization for Animal Health (OIE), the World Bank, the World Economic Forum and several other international organizations recognized AMR as a serious public health threat and global priority [1,9–13]. As the world is moving toward the post-Millennium Development to Sustainable Development Goals (SDGs) [14,15], food safety is a critical element to improve and strengthen global health, security and ensure sustainable development [12]. This paper presents the challenge of AMR through the lens of food safety. First, it highlights foodborne illness as serious food safety and public health issue; second, it underlines the multisectorial and multidimensional implications of the emergence and spread of AMR in the farm-to-plate continuum on the SDGs of food security, public health, animal health and welfare, the environment and climate, and socioeconomic development; and finally, it suggests a global strategy to ensure food safety along with sustainable development.

## Foodborne illness as an important food safety & public health issue

Food safety is an area of global public health priority and a vital element to achieving several SDGs such as those pertaining to poverty, hunger and promoting health and well-being. Food is considered unsafe when it is likely to physically harm the consumer as a result of damage, deterioration and presence of biological or chemical substances [16]. It can become unsafe at different stages in the farm-to-plate continuum, either during the production, distribution, retail/sale or preparation and consumption. Unsafe food can cause several health problems ranging from acute or chronic food poisoning or foodborne illnesses, reproductive and developmental concerns, to AMR, cancers and deaths [17]. Antimicrobial use at production level leads to food safety issues associated with the presence of antibiotic residues [17], ARB [3] and ARGs [3] in food animals and products, whereas the lack of adequate transportation and storage facilities as well as limited hygiene practices lead to breach of food safety and the deterioration of food integrity during the transport, storage and processing, respectively [16].

Emergence of AMR in the pre- and post-harvest systems presents a serious risk of contamination or infection directly by ARB and ARGs for farmers, agricultural practitioners, abattoir workers, food handlers and their associated contacts as well as consumers at the end of the food chain [16]. Foodborne illnesses are among the main public health consequences associated with unsafe food and water, particularly in infants, young children, pregnant women, elderly and people who have weak immune systems and are at high risk of acquiring and dying from foodborne illnesses [5,18]. Foodborne illness ranges from mild and self-limiting diseases including diarrheal episodes, vomiting and nausea, to debilitating and life-threatening consequences such as neural and brain disorders, kidney and liver failures, disabilities, cancers and deaths [5,18]. Undernourished infants and children are more prone to develop more severe forms that often lead to premature deaths [5,18].

According to the WHO, foodborne illness caused approximately 600 million of cases with 420,000 premature deaths in 2010 [19]. The global burden of food safety issues associated with foodborne illnesses is unequally distributed and differs from geographical location, income levels, healthcare infrastructures, diets and local conditions. By undertaking a systematic review of literature, Ao *et al.* demonstrated that globally, around 3.4 million cases of invasive non-typhoidal *Salmonella* infection occurred from January 1990 to December 2012, with 681,316 deaths reported [20]. The study revealed that Africa had the highest incidence (227 cases per 100,000 population) and number of cases (1.9 million cases) annually with infants, young children and young adults being the most affected [20]. Resistant shiga-toxin and entero-hemorrhagic producing *Escherichia coli*, especially serogroups O157, O26, O91, O103, O111, O128 and O145, were similarly associated with severe foodborne gastroenteritis across the world [21]. Majowicz *et al.* revealed that shiga toxin-producing *E. coli* was involved in 2.8 million acute illnesses every year with 3,890 cases of hemolytic uremic syndrome, 270 cases of end-stage renal diseases and 230 deaths being reported from January 1990 to April 2012 in ten out of 14 WHO subregions [22]. More specifically, in South East Asia, approximately one million children under 5 years of age die every year from diarrheal episodes due to consumption of contaminated food and water [16,23]. Likewise, foodborne illnesses claim around 200,000 deaths annually in Nigeria with 124,400 being children under 5 years old [24].

In a staggering contrast, a report from the Foodborne Diseases Active Surveillance Network (FoodNet) of CDC’s Emerging Infections Program conducting active and population-based surveillance for laboratory-diagnosed infections revealed that over a 4-year period (2015–2018), 25,606 cases of infection, 5893 hospitalizations and 120 deaths were caused by pathogens transmitted through food with the principal pathogens being *Campylobacter* spp. (19.5%), followed by *Salmonella* spp. (18.3%) and shiga toxin-producing *E. coli* (5.9%) [25]. The emergence of resistant foodborne illnesses will thus considerably increase morbidity and mortality around the world, albeit the problem will be more severe in developing countries where AMR in the food chain is already considerably neglected regardless of the high prevalence of ARB and ARGs reported in food animals and food products [26], and due to limited conditions prevailing in the food production industry, suboptimal hygienic conditions, unsafe water used for cleaning and cooking, and poor food handling [18].

Despite foodborne intestinal diseases due to *E. coli*, extra-intestinal illnesses associated with animal-originating resistant bacteria are recently gaining considerable attention, with foodborne urinary tract infections (UTIs) representing the major paradigm shift in the understanding of these illnesses [27]. ARB from contaminated food products can transiently colonize the human GI tract that becomes a reservoir for subsequent infections [27]. Similar genetic fingerprints have been detected in geographically and temporally matched UTI cases and *E. coli* from food. Jakobsen *et al.* revealed that foodborne *E. coli* were not only genetically associated with UTIs in humans but were also responsible of UTIs *in vivo*, with 13 foodborne phylogroup B2 of *E. coli* strains causing UTIs and pyelonephritis in a murine UTI model [28].

Altogether, the emergence and spread of ARB and ARGs in the farm-to-plate continuum will likely hinder the success of infection and prevention control measures and antimicrobial stewardship programs implemented in communities and hospitals [6,29]. The breach in the food safety barrier due to resistant bacteria of animal origin threatens in parallel, health policies implemented to contain sustainably noncommunicable diseases including diabetes mellitus, cardiovascular, pulmonary and heart diseases, and communicable diseases such as HIV-AIDS, tuberculosis, malaria as immune-compromised patients suffering from these pathologies will be most affected [29]. As consequences of these and other likely damaging effects of the emergence and spread of AMR in the farm-to-plate continuum, the SDG 1 and 3 of ending poverty and ensuring healthy lives and promoting well-being would be difficult to achieve without effective antibiotics [29,30].

In spite of all these concerns, food safety is still a veiled and often unheeded problem worldwide. The WHO consequently devoted its 2015 World Health Day to food safety, under the theme ‘From Farm-to-Plate, Make Food Safe,’ to invigorate decision-makers and all stakeholders to implement effective and efficient measures that will improve safety of food on farms, factories, slaughterhouses, supermarkets, street vendors and at kitchens [5].

## AMR in the farm-to-plate continuum at the edge of United Nations’ sustainable development goals

**Figure 1.**
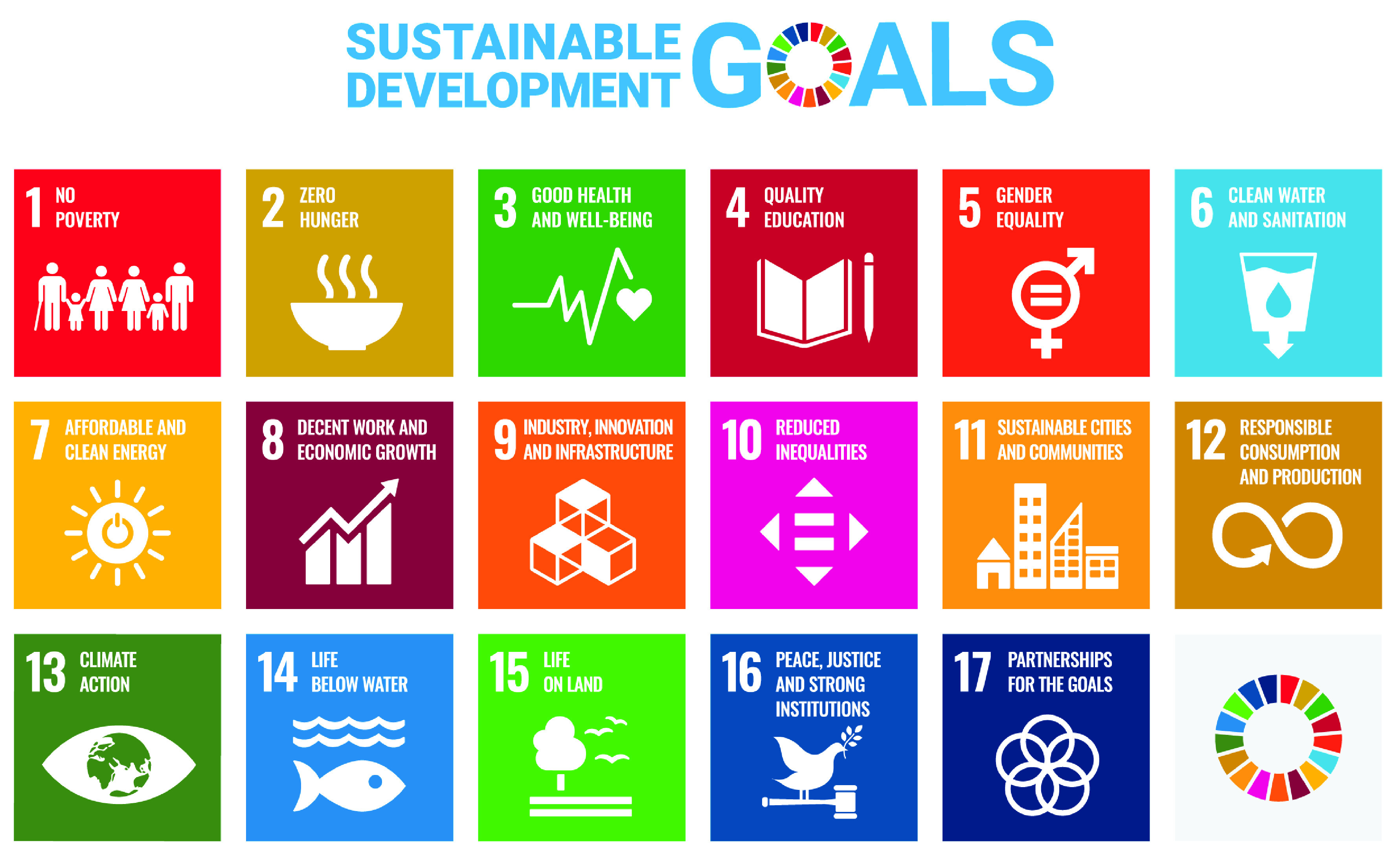
Chart of 17 Icons of UN Sustainable Development Goals.

Any breach in the food safety barrier leading to the emergence and spread of ARB and ARGs has multisectoral implications and threatens to reverse decades of human and animal health improvements globally. Food security, animal health and welfare, climate and environmental health as well as socioeconomic development are likely to be impacted by the emergence of AMR in the farm-to-plate continuum (Figure 1).

### Food security implications

Food security is achieved ‘when all people, at all times, have physical and economic access to sufficient, safe and nutritious food that meets their dietary needs and food preferences for an active and healthy life’ [31]. Globally, food animals are significant sources of food, and the food production industry is an important contributor to the world economy [32,33]. Meat, milk and eggs are the major commodities produced from classical food animals such as cattle, buffaloes, pigs, poultry, sheep and goats, whereas in aquaculture, fish, crustaceans and molluscs are the foremost [34,35]. The annual consumption of meat is around 80 kg/person in high-resource countries while it is about 10 kg/person in low- and middle-income countries (LMICs) [36]. In 2010, 296 million tonnes of meat were produced worldwide, with 37% being pig meat, 33% poultry meat, 23% beef or buffalo meat and 5% goat or sheep meat [36]. The same year, 69 million and more than 700 million tonnes of eggs and milk were produced, respectively; and aquaculture contributed with approximately 60 million tonnes of fish [36]. The UN SDG 2 promotes end of hunger, achievement of food security, improved diet and sustainable agriculture by 2030 [15, 76].

However, nowadays, 700 million people living in rural areas are still living under extreme conditions of poverty. Approximately a third of food produced is wasted or lost, leading to unnecessary pressure on Earth’s resource and losses for agricultural practitioners [37], while around two billion people suffer from micronutrient deficiencies and 800 million are chronically hungry worldwide [37]. The situation is likely to worsen propelled by the 2.3 billion persons increase in global population during the next 35 years. Indeed, according to the UN Department of Economic and Social Affairs report, the current global population of 7.3 billion is estimated to reach 8.5 billion in 2030, 9.7 and 11.2 billion by 2050 and 2100, respectively [38]. This drives the world toward a threefold challenge: to match this unprecedented demand on animal and crop proteins from a bigger and more affluent population to its supply, do so in ways that are environmentally and socially sustainable, and ensure that the world’s poorer people are continually food secure [39].

When food supplies become insecure, people lean toward less healthy diets and consume more unsafe food containing chemical and microbiological hazards and posing health risks [5] as food safety and security share a fundamental basis and are inextricably connected to one to another with feedback effects [31,35]. Van Boeckel *et al.* reported that changes from extensive farming systems toward large-scale intensive and industrialized production will be observed, with evident upsurge of negative conditions such as overcrowding, intensive human contacts with animals and environment, as well as considerable antimicrobial use in the food production industry that will lead to increased AMR in agriculture, and thereby in the farm-to-plate continuum [40]. Unsafe food due to the emergence and spread of ARB and/or ARGs in food animals and products leads to a vicious cycle of worsening health including malnutrition and foodborne illnesses that endangers the nutritional status of most vulnerable as well as the achievement of global food security as it may lead to chronic or transitory food insecurity across the world [31,35].

In fact, the global spread of ARB and/or ARGs in the farm-to-plate continuum may cause price instability *viz*. increase of food costs [1,39], as well as devastation of whole herds or flocks when resistant outbreaks occur in several production systems [41]. It will thereby lessen access to safe food to poorer people and significantly aggravate hunger [1]. Hunger hotspots may also emerge particularly in developing nations due to extensive emergence and persistence of ARB and ARGs in food animals and products, and protract the crisis [1,39]. Half a billion people located in more than 20 countries are already affected by hunger and this could be aggravated by AMR [1,39]. This implies that the extensive use of antimicrobial medicines in farming practices will threaten the achievement of UN’s SDG 2 of eliminating hunger in our lifetime by 2030. Similarly, it will impede the eradication of all forms of poverty (SDG 1), endanger the achievement of global well-being (SDG 3) and jeopardize sustainable consumption and production (SDG 12).

Building efficient, inclusive and resilient food systems is needed for effective management of food security [37], as any efforts addressing food security or sustainable consumption and production will have an impact on food safety and vice versa [39]. Development of sustainable food production systems where high productivity is not over-reliant on antibiotic use, is of crucial importance to double the quantity of animal and crop proteins and reach the SDG 2 [1,15,35,39]. The Rome Declaration on Nutrition (2014) and World Food Summit (2012) already recognized that food systems must contribute to address and prevent infectious diseases, and curb the worldwide dissemination of AMR [35]. It endorsed a Framework for Action recommending actions on food safety and AMR: to raise awareness among all stakeholders on this threat, implement adequate and comprehensive measures for its containment, and develop and implement national guidelines on judicious use of antibiotics in food animals, in line with international standards of competent supranational organizations [35].

Achievement of global food security would thus require not only comprehensive sustainable consumption and production, but mainly, sustainable access to safe food worldwide, thereby confirming food safety as one of the key elements to achieve world’s food security [29,35]. Implementation of appropriate policies and activities to fight AMR are further of crucial importance to ensure food security for current and future generations [30,42]. Although the FAO recently called for a One Health and food chain approach to respond to the increasing threat of resistance in the world’s food production systems, eradicate hunger and rural poverty, considerable public health strategies and activities are still required to ensure consumers’ health through food safety.

### Implications for animal health & welfare

AMR crosses food safety and security to reach animal health and welfare. Several reports on the consequences of AMR emergence and spread in the food chain relate the danger of ARB and ARGs transmission to people, and thereby its potential public health implications [8,13,41]. However, the repercussions of AMR in animals are not merely limited to human health and it has been reported that the emergence of ARB and ARGs in the food chain put animal health and welfare at high risk and will subsequently have adverse repercussions on human livelihoods and food security.

Antimicrobials are critical to treat and prevent diseases in animals (as with humans) and are essential tools in maintaining animal health and welfare [8,13]. However, without timely and responsible use of effective antimicrobials, a resistant infectious disease could rapidly spread on-farm with direct and indirect negative impacts on animal health and welfare, endanger health of herds/flocks and safety/security of food [8,13].

For instance, soft tissue and surgical site infections in animals could, if left untreated due to inefficiency of antimicrobials, be life-threatening or lead to euthanasia of infected animals for welfare reasons [41]. Enteric and respiratory diseases are among the most significant in several animal species such as pigs and poultry, and mastitis is frequent in animals reared for milk production, including cows, goats, sheep and buffaloes [41]. These diseases are highly contagious and therefore more problematic when associated with resistant bacteria in farming systems where animals are kept in large and overcrowded groups, as they will cause drug-resistant infections of whole herds/flocks and thereby result in suffering of the animals [41]. To alleviate their implications, antimicrobials are used therapeutically for the treatment of sick animals, thereby preventing suffering and, as such, contributing to the welfare of animals, food safety and security, and productivity [8,13]. This aspect has as-yet received minimal or no scientific attention and, unlike in humans where the WHO published a global report on AMR [43], the worldwide burden of resistance on animal health is unknown and no report from the OIE has mentioned it [41].

The main way to minimize the levels of antimicrobial use in animals is to prevent infections and ensure good animal care, as on-farm activities directly affect the level of medications required. The key elements are biosecurity measures (good agricultural and hygiene practices) and immunization that prevent the emergence and transmission of infectious diseases, ARB and ARGs, along with commercial incentives and disincentives, and, a legal framework regulating antibiotic use in animal sector. Experiences of some countries such as Denmark, Sweden, Norway and The Netherlands confirm that antimicrobial use in animals can be substituted with alternative measures including vaccines, good husbandry practices and biosecurity measures, without disruption of the productivity. This does not mean that antimicrobials should not be used at all, but rather, that it is crucial to maintain antimicrobial effectiveness for treating very sick animals, thereby reducing suffering, ensuring good welfare and top-class food safety for the consumers.

Also, given the fact that AMR has reduced the therapeutic arsenal for several animal diseases some antibiotics are no longer proposed as first-line drug and there are only few alternatives remaining for their management due to the widespread resistance. Penicillin for instance, which has been used for the treatment of mastitis caused by *S. aureus* in cattle since the 1950s, is currently not an empirical first choice for this condition. Emergence of penicillin and tetracycline resistance in *Mannheimia haemolytica* (*M. haemolytica*) and *Pasteurella multocida* (*P. multocida*) responsible for pneumonia in calves make it difficult to use these drugs as first-line treatment in some part of the world [41]. Similarly, resistance in *E. coli* causing enteritis in young pigs has worldwide ousted trimethoprim-sulfamethoxazole as appropriate first therapeutic option [44].

Swine dysentery caused by the spirochete *Brachyspira hyodysenteriae* (*B. hyodysenteriae*) is a serious enteric infection for suckling pigs that usually affects a large proportion of animals within a herd and persists with recurring outbreaks causing animal suffering and economic losses attributed to mortality, limited feed conversion and retarded growth [41]. Resistance to antimicrobials previously used in the management of swine dysentery, including tylosin and lincomycin, is actually widespread, and nowadays, pleuromutilins are recommended. However, resistance to pleuromutilins has also been detected, making the management of swine dysentery difficult with serious constraints on the production of the farm where outbreaks of resistant *B. hyodysenteriae* emerge [41].

**Figure 2.**
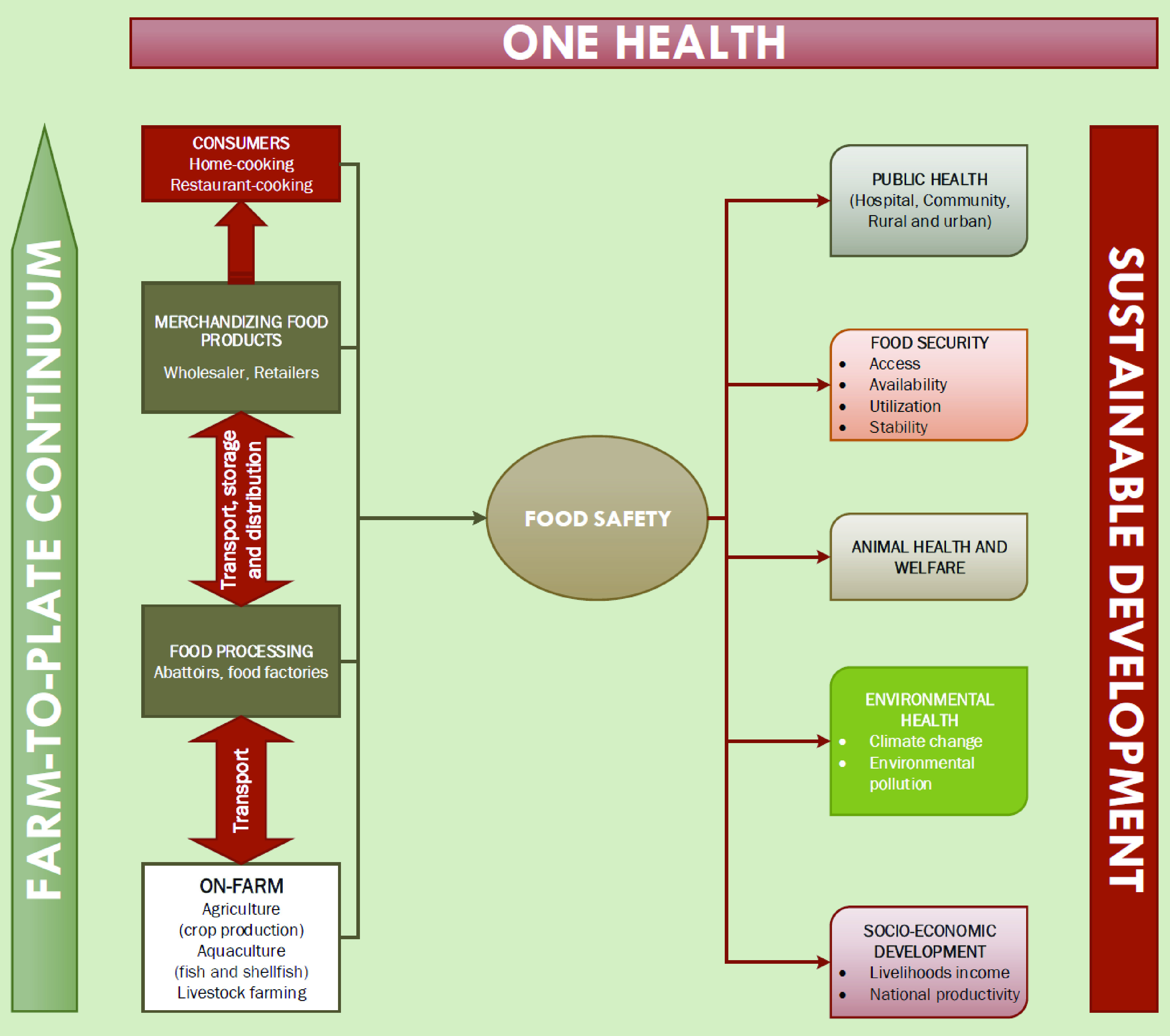
Multidimensional repercussions of the emergence of antimicrobial resistance in the farm-to-plate continuum.

The MRSA clonal complex 398 emerged in the last decade in pigs, and asymptomatically colonized various food animals. It has now been detected in milk and associated with mastitis in dairy cows with concomitant resistance to several antibiotics including tetracycline, gentamicin, trimethoprim-sulfamethoxazole and erythromycin [45]. This implies that should MRSA strains become more prevalent in mastitis, for instance, there will be limited or no antimicrobials available for its management [41]. Although such scenarios could certainly be managed in single herds through depopulation, cleaning and subsequent restocking under biosecurity measures, emergence and spread of multiresistance in endemic bacteria such as *M. haemolytica* and *B. hyodysenteriae*, in indicators like *E. coli* and *Enterococcus* spp., and emerging pathogens such as MRSA clonal complex 398, would have serious implications for productivity (especially in large-scale intensive production systems), food security and safety regardless of country-income, thereby threatening achievement of SDGs 1, 2, 3 and 12 (UN, 2015; Figure 2). Preserving the effectiveness of the current therapeutic arsenal is therefore crucial to ensure our ability to alleviate the threat posed by ARB and ARGs for animal health and welfare, as well as public health.

### Implications for socioeconomic development

In its 2013 annual report on global risks, the World Economic Forum concluded that ‘arguably the greatest risk ... to human health comes in the form of antibiotic-resistant bacteria. We live in a bacterial world where we will never be able to stay ahead of the mutation curve. A test of our resilience is how far behind the curve we allow ourselves to fall’ [10]. This has been confirmed by the AMR review which stated that a cumulative global economy loss of US$100 trillion over the 35 next years will be attributable to AMR if considerable efforts are not sustainably implemented [4]. In addition, the World Bank estimated that the annual cost of AMR could be as high as those of the 2008’s global financial crisis and that LMICs would be most affected with the largest economic shortfalls in economic growth [9]. In fact, LMICs are estimated to face a total productivity loss of around US$95 billion yearly owing to unsafe food with the cost of treating foodborne illnesses being projected at US$15 billion [19]. The total productivity loss associated with foodborne illnesses in LMICs is estimated to cost US$95.2 billion per year, and the annual cost of treating them is estimated at US$15 billion. Although difficult to quantify, other costs include company sales and losses of farm, foregone trade income, the health repercussions of consumer avoidance and the environmental burden of food waste. The majority of the health and economic burden could be avoidable through simple preventive measures, behavioral changes and investments adopted in the farm-to-plate continuum [19].

Furthermore, financial losses that will occur due to higher mortality directly associated with resistant infections, and indirectly through reduced feed conversion, decreased growth and production, as well as premature culling of dairy cows and breeding animals, will result in higher costs of commodities derived from animal food production and ultimately for the end consumer [41]. AMR, therefore, negatively impacts agricultural practitioners and food production industry, as the absence of effective antibiotics to treat sick animals will irreversibly damage food productivity, leads to loss in consumers’ confidence in the products, thereby reducing benefits [8,37,46].

In the lens of the consumer, substantial medical expenses, together with absenteeism at work and school that is prejudicial for the society will also be attributed to resistant foodborne infections [47,48]. US National data of seven foodborne pathogens reported losses between US$5.6 and 9.4 billion in absence at work and medical expenses, whereas in the European Union, annual healthcare costs attributed to Salmonella infection alone were estimated at €3 billion. The cost of 11,500 daily cases of foodborne infections was estimated at AU$2.6 billion annually in Australia. Based on 2011 CDC data, Scharff estimated at US$1626 and 77.7 billion the average cost per case and annual cost of foodborne illness, respectively [49].

Moreover, consequences related to food contamination will drain the national economy as country economic losses associated with resistant foodborne infections will range from increase of national medical expenses, outbreak investigations and food recalls. In 2011, an outbreak due to enterohemorrhagic *E. coli* and linked with contaminated fenugreek sprouts emerged in Germany and spread in height countries in Europe and North America, with 53 deaths reported. This outbreak caused US$1.3 billion losses for German farmers and industries and up to US$236 million in emergency aid payments to 22 European Union member states [5]. The same year, an outbreak linked to a strain of *Salmonella* resistant to four antibiotics and originating from ground turkey caused 136 cases with one death and recalling of 36 million lbs of ground turkey in the USA [50].

The 2016 World Bank report revealed that output and trade in food animals and products are especially vulnerable to AMR effects not merely because of reduced productivity associated with resistant infections but also due to international trade disruption in the wake of disease outbreaks [9]. The report estimated that decline in global food production could range between 2.6–7.5% of food products and LMICs will be the most affected with 11% loss in a simulation of worst AMR impact scenario [9]. This suggests that if nothing is done to significantly address this threat, the socioeconomic development of several countries, particularly those in the developing world that relies mainly on agriculture and food production, will be seriously impeded, along with the achievement of some world’s SDGs. For instance, SDG 1 of eradicating poverty by 2030 would be difficult to reach as the number of poor people will be 8 million in an optimistic and up to 24 million in a pessimistic scenario, and the majority of these increases will occur in LMICs [9]. To a further extent, elimination of hunger, achievement of well-being for all, ensuring equitable education, sustainable management of water and sanitation for all as well as sustainable economic growth, and sustainable consumption promoted under SDGs 2, 3, 4, 6, 8 and 12 (Figure 2), respectively, may likely not be accomplished by the same period.

### Implications for environmental health & climate change

#### Environmental health

The use of antibiotics for prevention, treatment and growth promotion purposes in animal husbandry and fish farming as well as in crop production enables their release in natural ecosystems [51,52]. A large fraction (30–90%) of these antibiotics are not transformed into inactive compounds, retain their biologically active form and enter either to waste water treatment plants (WWTPs) or directly into groundwater or soils with one of three fates: absorption to sewage sludge [53]; biodegradation [54,55] or persistence unchanged in the effluent [54]. The dissemination of antibiotics, antibiotic residues, as well as ARGs and ARB excreted via food animal waste leads to ‘environmental pollution’ and therefore, establishes the environment as an important reservoir for the emergence and spread of AMR across the farm-to-plate continuum [54].

In fact, the aquatic environment can serve both as natural AMR reservoir and route for the entrance in the food chain since aquatic bodies such as rivers, lakes, streams and even coastlines are generally end points receiving effluents from WWTPs and agricultural runoff, and increasing thereby the level of ARB and ARGs in natural ecosystems [56,57]. A critical review by Williams *et al.* highlighted oceans as the largest reservoir of ARGs in the environment, with 28% of known genes including clinically relevant and unknown genes [56]. The authors also reported that the incidence of tetracycline resistance was twice as high in coastal runoff compared with forested area runoff as well as around 35% of β-lactam ARGs (bla_TEM-1 + SHV12_ and bla_CTX-M-15_) in environmental *Enterobacteriales* [56]. This poses a serious public health risk as accidental ingestion of ARB- or ARG-containing water may occur during bathing or recreational activities. Considering that 1.8 billion people, especially in developing countries, do not have access to safe drinking water, the high rate of ARGs reported in water surfaces could drastically increase morbidity and mortality due to resistant waterborne bacteria [58].

In addition, ARB and ARGs spread off a large livestock operation, termed confined animal feeding operation, by several routes including via manure applied to land as fertilizer, from lorry transporting animals, wind leaving farm/abattoir facilities or even via flies or beetles attracted to the dung, which can pick up and spread ARB. A recent study of the South Platte River in the USA, found that ARGs coding for resistance to sulfonamides were 10,000-times superior in river sediments downstream compared with those upstream from larger feedlots (ones with 10,000 cattle). The same study further revealed that these same ARGs were 1000-times higher from sewage treatment plants releasing ten million gallons of effluent per day compared with pristine sediments [59]. Once these pollutants of animal origin escape the farm, genetic exchanges of resistance mechanisms with other bacteria, even those that have never been exposed to antibiotics can ensue. Consequently, farm soils, manure, waste streams and WWTPs have been acknowledged as ‘hot spots’ of antibiotic pollution and dissemination of AMR in the food chain, with antibiotic residues, mobile genetic elements, ARGs and ARB being detected across the world in these ecological niches [60,61]. Horizontal gene transfer has then been documented in these niches as well as in rivers, lakes and wildlife animals, and are involved in microbial communities containing various levels of ARB and ARGs [2,51,52].

How the environmental pollution contributes to the selection of resistant bacteria and the exacerbation of the environmental resistome (assortment of genes that are able to confer ABR when expressed in a susceptible organism) elements is highlighted by a study from Huang *et al.* on 4767 commensal isolates originating from fish, feed and water of an aquaculture farm, which revealed that up to 80% were resistant to antibiotics with feed showing the highest levels upon real-time or quantitative PCR [62]. The dissemination of these materials onto soil increases AMR emergence and dissemination risks to: wild animals; crops, vegetables and fruits; surrounding water surfaces and groundwater; farm workers; and air and dust particles. Azanu *et al.* reported that carrot and lettuce, two vegetables that are frequently eaten raw, were associated with sub-inhibitory concentration of antibiotics (tetracycline and amoxicillin) upon water irrigation [63]. When recognizing that around 70% of global freshwater of agricultural production is based on resources from rivers, lakes and groundwater and is required to produce 20% of global foods [64], it is likely that ARB and ARGs spread to food animals and products, fish, shellfish, vegetables and water to ultimately reach the consumers and thereby, enhancing the public health risks associated with the presence of AMR in the farm-to-plate continuum [51,57].

Besides, animal waste is used throughout the world and particularly in developing countries as fertilizer in crop lands, and feeding of fish and shellfish in aquaculture. The persistence and changes in the resistome [65] of sludge or manure after anaerobic digestion or composting gained high interest worldwide and land spread of composted sludge has been associated with spread of ARGs in the soil and wider environment [66]. In keeping with this, Calero-Cáceres *et al.* reported an elevated prevalence of ARGs after digestion of sludge and suggested that agricultural use of sludge-harboring ARB could significantly contribute to the spread of AMR in the environment [67]. In addition, Xiao *et al.* identified 16 types of ARGs corresponding to 110 ARG subtypes encompassing major resistance mechanisms and with multidrug-resistant genes (e.g., gene-encoding efflux) being the most common type detected (range: 38–47.5%) in all samples in China [68]. Udiko-kolic *et al.* reported that application of manure as fertilizer to agricultural soil led to emergence and dissemination of AMR, even when the animals that produced the manure were not treated with antibiotics [69]. The authors further concluded that enrichment of resident soil bacteria harboring AMR elements is favored by manure fertilization [69]. All these confirm that farm environments, especially soil and water, may contribute to the spread of AMR along the farm-to-plate continuum. The environmental pollution is of particular concern as ARB and ARGs may spread through plant, crop production and wildlife, and revert back to animals and humans, thereby increasing the risk of transmission of foodborne infections and pose food safety and public health risks, particularly in developing countries due to the limited access to clean water and precarious sanitary conditions.

However, the impact of environmental pollution goes beyond selecting resistant mutants, favoring the acquisition of ARGs via horizontal gene transfer and food safety and public health threats, to the enrichment of the pool of intrinsically resistant microorganisms and reduction of susceptible ones in the environmental microbiota [54]. Cyanobacteria, for instance, that are responsible for more than a third of CO_2_ fixation and total free O_2_ production are naturally susceptible to antibiotics, and there is currently no evidence that this population is suffering the impact of antibiotic pollution. However, the elimination of cyanobacteria as a result of this pollution might imbalance the micro-biosphere and have impact on climate change [54]. The AMR environmental pollution, thereby, threatens achievement of SDGs 2, 3, 6, 12 and 15 promoting end of hunger, healthy lives and well-being, availability and sustainable management of water and sanitation for all, sustainable consumption and production as well as sustainable use of terrestrial ecosystem, respectively. It also endangers global actions undertaken in the mitigation of climate change.

#### Climate change

Food animal production and its environmental footprints is recognized as great contributor to climate change [37,64,70,71]. Indeed, after fossil fuels, animal agriculture is the second largest contributor of human made greenhouse gas (GHG) with deforestation, water and air pollution, and biodiversity losses being the main consequences [71,72]. A heavy strain on Earth’s finite resources such as water, land and energy is caused by animal agriculture in order to accommodate the world’s 70 billion animals annually raised to feed the human population. As such, around 60% of global freshwater and a third of the planet’s ice-free land and global grain production are dedicated to growing of food animals [37,64,70,71].

With the world’s population projected to reach 9.7 billion by 2050 [38], the FAO estimates that the demand of animal proteins and crops, both through population growth and economic development, will require as much as a doubling the global food and agriculture production over the same time period [37]. The consumption of meat and dairy products is projected to rise by 76 and 64%, respectively, thereby, increasing the resource load from the food production industry [33,37,64].

The production and distribution of quality, nutritious and safe food for the expected 9 billion population by 2050, without destroying Earth’s finite resources, including antimicrobial substances, represent a multifaceted challenge that lacks a single solution [33,39]. The environmental repercussions of agricultural industrialization/expansion increase with the global food demand and this has been confirmed by a recent modeling study, which revealed that meeting the unprecedented global demand on animal proteins relies on the agricultural evolution. If greater agricultural industrialization continues, around 1 billion ha of land would be cleared globally by 2050, with concentration of GHG emissions reaching approximately 3 Gt y^-1^ and azote use approximately 250 Mt y^-1^ by the same year [33].

As one of the foremost users of natural resources and thereby contributor to climate change, the food animal production sector needs to address its environmental footprints [70–72]. The sector currently faces the significant challenge to reduce its GHG emissions with the large part being caused by methane released from enteric fermentation and partly from animal manure [70,72], while responding to a substantial demand for animal and crop proteins [33]. This situation led farmers and food producers to favor and implement intensive agricultural practices that are over-reliant on antimicrobial medicines [40] that, besides threatening human, animal and environmental health with the emergence of ARB and ARGs, further exacerbates climate change and undermines the considerable efforts undertaken to combat it as depicted in SDGs 13 and 15 [15].

As food safety is intricately linked to sustainable consumption and production [34,39], AMR in the food chain may cause an imbalance leading to environmental issues and jeopardizing achievement of UN’s SDGs 12 and 13 [15]. This is confirmed by Hammer *et al.*, who revealed that antimicrobial use in food animals increases GHG emissions from cow dung. The authors showed that antibiotics, especially tetracycline used in this study, boost methane production in cowpats by favoring ARB and methane-producing organisms in the digestive systems [73]. The authors reported that manure of cattle feed with tetracycline produced 1.8-times more methane, a potent and principal global warming gas, than normal manure, while their belches produced far more methane than their manure did [73]. The challenges of increased food production are magnified by its potential impact on environmental sustainability and resource availability, which will require entirely new approaches, public–private collaboration, scientific discovery and translation of results into effective strategies and policies for decision-makers.

## Global strategy to ensure food safety & sustainable development

Containing AMR requires a global approach combining multifaceted and comprehensive actions within the One Health approach, with a strong overarching goal to ensure food safety, sustain food security, combat communicable and noncommunicable diseases successfully, as well as preserve antimicrobial effectiveness for future generations [4]. In keeping with this, a better use of antimicrobial substances in food production is needed while substantially preventing and monitoring the transmission of resistance already present in the farm-to-plate continuum as containing it throughout this continuum will substantially lessen multisectorial implications associated with this issue ([1]; Table 1 & Figure 2). Interdependent and inter-related problems such as AMR need integrated and global solutions [74] as per the examples of Denmark, Sweden and Norway where levels of antibiotic use and resistance are the lowest in the world, a situation partly due to the effective containment of AMR concomitantly in humans and animals.

**Table 1.** Summary of implications of antimicrobial resistance in the farm-to-plate continuum.

Implications of AMR	Direct impacts	Indirect impacts	SDGs affected
Food security	Unsafe food Increased hunger and malnourishment	Increased morbidity and mortality Increased poverty	SDG^†^ 1, 2, 3 and 12
Animal health and welfare	Therapeutic failure Increased animal morbidity and mortality Threat to animal well-being	Increased cost of food production Increased hunger Endangered human health	SDG 1, 2, 3 and 12
Public health	Intestinal and extra-intestinal foodborne illness	Endangered IPC^‡^/ASP^§^ Increased cost of hospitalization and medication	SDG 1 and 3
Environmental health	Environmental pollution Unsafe water Change of environmental microbiome	Increased hunger Threatened sustainable consumption and production Endangered sustainability of Earth’s finite resource Endangered human and animal health	SDG 2, 3, 6, 12, 15
Climate change	Increased GHG emissions Deterioration of natural resources	Endangered public health, food security and climate change actions	SDG 2, 3, 6, 12, 13, 15
Socioeconomic development	Increased poverty Drain on national economy	Increased hunger Threatened education and well-being Endangered policies and actions on sustainability	SDG 1, 2, 3, 4, 6, 8, 12

† Sustainable development goals.

‡ Infection, prevention and control.

§ Antimicrobial stewardship programs.

AMR: Antimicrobial resistance; GHG: Greenhouse gas.

A multi-pronged strategy has been proposed to ensure food safety as it is a collective responsibility requiring collaboration among the government, food-producing industries and the public across the farm-to-plate continuum (*viz*., from farmers and food manufacturers to food-handlers and end consumers). Decision-makers should acknowledge food safety a public health priority, to ensure that all stakeholders along the whole farm-to-plate continuum, from the food producers and suppliers to the consumers, operate responsibly to preserve the safety of food while ensuring food security [75]. Farmers should, for instance, be advised to implement effective biosecurity measures to prevent emergence of AMR and on-farm contamination with ARB and ARGs or external dissemination when contamination occurs, whereas food-handlers and consumers are advised to make use of WHO’s tool ‘Five Keys to Safer Food’ when cooking [75]. Decision-makers should further implement and maintain adequate and sustainable food systems and infrastructures in order to manage appropriately food safety risks throughout the entire farm-to-plate continuum. The development of specific and achievable goals, reinforcement of political will, mobilization and allocation of resources, financial support to reinvigorate antibiotic pipeline and alternatives to antibiotics, and agreement on a responsible mechanism for global collective action on this threat are further required [74].

## Conclusion & key recommendations

The emergence and spread of AMR in the farm-to-plate continuum is a food safety issue with public health implications just being the tip of the iceberg. Not considered are the manifold implications on food security, animal health and welfare, socioeconomic development, environment and climate change, which make the emergence and spread of AMR in this continuum among the keystones of this global disaster. In this regard, emergence and spread of AMR in the farm-to-plate continuum should not merely be considered a food safety issue but recognized as a grave barrier in the accomplishment of UN’s SDGs. Policy makers and governments should invest smartly in food safety, heighten awareness to engage effectively with all stakeholder, especially consumers and implement appropriate preventive measures in the farm-to-plate continuum.

## Future perspective

This perspective emphasizes that AMR in the food chain is a silent pandemic threat requiring holistic actions and sustainable political will and commitment. It further reveals that the manifold repercussions of AMR in the food chain extend beyond reduced productivity, benefits or health risks and food safety to reach food insecurity, higher healthcare costs, drain on national and global economies as well as worsening climate change. Upcoming efforts for containment of AMR in the farm-to-plate continuum should focus on a collaborative and holistic One Health approach that considers not only the problem itself but also all upstream and downstream factors associated with it. As with threats posed by the climate change, where positive results of interventions are already evident, sustainable efforts endorsed, coordinated and monitored by the United Nations in line with One Health approach, and involving WHO, FAO, OIE, UN Environment Programme (UNEP); the UN Children’s Fund (UNICEF), UN Development Program (UNDP), UN Educational, Scientific and Cultural Organization (UNESCO), the World Bank and other multilateral agencies should be implemented to contain AMR from farm-to-plate, as part of a global coordinated plan.

**Executive summary**

- Antimicrobial resistance (AMR) in the food chain is a silent pandemic threat with public health implications just being the tip of the iceberg.
- Food safety is an area of global public health priority and a vital element to achieving several sustainable development goals such as those pertaining to poverty, hunger and promoting health and well-being.
- Any breach in the food safety barrier leading to the emergence and spread of antibiotic-resistant bacteria and antibiotic-resistant genes has multisectoral implications and threatens to reverse decades of human and animal health improvements globally.
- Emergence of AMR in the pre- and post-harvest systems presents a serious risk of contamination or infection directly by antibiotic-resistant bacteria and antibiotic-resistant genes for farmers, agricultural practitioners, abattoir workers, food handlers and their associated contacts as well as consumers at the end of the food chain.
- The manifold repercussions of AMR in the food chain thus extend beyond reduced productivity, benefits or health risks, and food safety to reach food insecurity, higher healthcare costs, drain on national and global economies as well as worsening climate change.
- Assessing the real implications of AMR is difficult, as such, the numerous ecological niches of the farm-to-plate continuum need to be integrally considered when addressing the effective containment of AMR, conservation of antimicrobials for current and future generations, as well as sustainable development by 2030.
- Implementing the One Health approach is essential as sustainable healthcare solutions for current and future generations cannot be achieved in isolation but require well-coordinated efforts locally, nationally, regionally and internationally.

## References

[1] The United Nations Food and Agriculture Organization (FAO). Status Report on Antimicrobial Resistance. FAO, Rome, Italy (2015).

[2] Holmes AH, Moore LSP, Sundsfjord A Understanding the mechanisms and drivers of antimicrobial resistance. Lancet 387(10014), 176–187 (2016). 26603922 10.1016/S0140-6736(15)00473-0

[3] Founou LL, Founou RC, Essack SY. Antibiotic resistance in the food chain: a developing country-perspective. Front. Microbiol. 7(1881), (2016). 10.3389/fmicb.2016.01881 PMC5120092 27933044

[4] O’Neill J. Tackling drug-resistant infections globally: final report and recommendations. In: Review On Antimicrobial Resistance. (2016).

[5] World Health Organization (WHO). WHO estimates of the global burden of foodborne diseases: foodborne disease burden epidemiology reference group 2007–2015. WHO, Geneva, Switzerland (2015).

[6] EFSA and ECDC. The European Union summary report on antimicrobial resistance in zoonotic and indicator bacteria from humans, animals and food in 2014. EFSA J. 14(2), 4380 (2016). 10.2903/j.efsa.2018.5182 PMC7009656 32625816

[7] Robinson TP, Bu PD, Carrique-Mas J Antibiotic resistance is the quintessential One Health issue. Trans. R. Soc. Trop. Med. Hyg. 110, 377–380 (2016). 27475987 10.1093/trstmh/trw048 PMC4975175

[8] The United Nations Food and Agriculture Organization (FAO). Drivers, dynamics and epidemiology of antimicrobial resistance in animal production. FAO, Rome, Italy (2016).

[9] World Bank. Drug-Resistant Infections: A Threat to Our Economic Future (Discussion Draft). World Bank, Washington, DC, USA (2016).

[10] World Economic Forum. Global risks 2013. : An initiative of the Risk Response Network. World Economic Forum (2013).

[11] The United Nations. Interagency Coordination Group on Antimicrobial Resistance. UN, NY, USA (2017).

[12] World Health Organization (WHO). Global Action Plan on Antimicrobial Resistance. WHO, Geneva, Switzerland (2015). 10.7196/samj.9644 26242647

[13] World Organization for Animal Health (OIE). The OIE Strategy on Antimicrobial Resistance and the Prudent Use of Antimicrobials. OIE, Paris, France (2016).

[14] Sachs JD. From Millennium Development Goals to Sustainable Development Goals. Lancet 379, 2206–2211 (2012). 22682467 10.1016/S0140-6736(12)60685-0

[15] The United Nations. Transforming our world: The 2030 Agenda for Sustainable Development. United Nations, NY, USA (2015).

[16] Prabhakar SVRK, Sano D, Srivastava N. Food safety in the Asia-Pacific Region: current status, policy perspectives and a way forward. : Sustainable consumption and production in the Asia-Pacific region: Effective responses in a resource constrained world. Institute for Global Environmental Strategies, Hayama, Japan, 215–298 (2010).

[17] Mensah SEP, Koudandi OD, Sanders P, Laurentie M, Mensah GA, Abiola FA. Antimicrobial residues in foods of animal origin in Africa: public health risk. Rev. Sci. Tech. Off. Int. Epiz. 33(3), 987–996 (2014). 25812221

[18] World Health Organization (WHO). Tackling antibiotic resistance from a food safety perspective in Europe. WHO, Europe, Copenhagen, Denmark (2011).

[19] Jaffee S, Henson S, Unnevehr L, Grace D, Cassou E. The Safe Food Imperative: Accelerating Progress in Low- and Middle-Income Countries. Series, AaF World Bank, Washington, DC, USA (2019).

[20] Ao TT, Feasey NA, Gordon MA, Keddy KH, Angulo FJ, Crump JA. Global burden of invasive nontyphoidal Salmonella Disease, 2010. Emerg. Infect. Dis. 21(6), 941–949 (2015). 10.3201/eid2106.140999 PMC4451910 25860298

[21] Ojo OE, Ajuwape AT, Otesile EB, Owoade AA, Oyekunle MA, Adetosoye AI. Potentially zoonotic shiga toxin-producing *Escherichia coli* serogroups in the faeces and meat of food-producing animals in Ibadan, Nigeria. Int. J. Food Microbiol. 142(1–2), 214–221 (2010). 20643488 10.1016/j.ijfoodmicro.2010.06.030

[22] Majowicz SE, Scallan E, Jones-Bitton A Global incidence of human shiga toxin–producing *Escherichia coli* infections and deaths: a systematic review and knowledge synthesis. Foodborne Pathog. Dis. 11(6), 447–455 (2014). 24750096 10.1089/fpd.2013.1704 PMC4607253

[23] Jahan S. Epidemiology of foodborne illness. : Scientific, Health and Social Aspects of the Food Industry. Valdez B (). InTech, Rijeka, Croatia (2012).

[24] Ajayi OA, Salaudeen T. Consumer food safety awareness and knowledge in Nigeria. Int. J. Food Safety 16, 17–24 (2014).

[25] Tack DM, Marder EP, Griffin PM. Preliminary incidence and trends of infections with pathogens transmitted commonly through Food — Foodborne Diseases Active Surveillance Network, 10 U.S. Sites, 2015–2018. MMWR Morb. Mortal. Wkly Rep. 68, 369–373 (2019). 31022166 10.15585/mmwr.mm6816a2 PMC6483286

[26] Founou LL, Amoako DG, Founou RC, Essack SY. Antibiotic resistance in food animals in Africa: a systematic review and meta-analysis. Microb. Drug Resist. 24(5), 648–665 (2018). 29683779 10.1089/mdr.2017.0383

[27] Singer RS. Urinary tract infections attributed to diverse ExPEC strains in food animals: evidence and data gaps. Front. Microbiol. 6(28), (2015). 10.3389/fmicb.2015.00028 PMC4316786 25699025

[28] Jakobsen L, Hammerum AM, Frimodt-Moller N. Detection of clonal group A *Escherichia coli* isolates from broiler chickens, broiler chicken meat, community-dwelling humans, and urinary tract infection (UTI) patients and their virulence in mouse UTI model. Appl. Environ. Microbiol. 76, 8281–8284 (2010). 21037306 10.1128/AEM.01874-10 PMC3008234

[29] Jasovsky D, Littmann J, Zorzet A, Cars O. Antimicrobial resistance- a threat to the world’s sustainable development. Upsala J. Med Sci. 121(3), 159–164 (2016). 27416324 10.1080/03009734.2016.1195900 PMC4967260

[30] Ardal C, Outterson K, Hoffman SJ International cooperation to improve access to and sustain effectiveness of antimicrobials. Lancet 387, 296–307 (2016). 26603920 10.1016/S0140-6736(15)00470-5

[31] FAO. The State of Food and Agriculture. FAO, Rome, Italy (1996).

[32] Smith J, Sones K, Grace D, MacMillan S, Tarawali S, Herrero M. Beyond milk, meat, and eggs: role of livestock in food and nutrition security. Animal Front. 3(1), 6–13 (2013).

[33] Tilman D, Balzer C, Hill J, Befort BL. Global food demand and the sustainable intensification of agriculture. Proc. Natl Acad. Sci. 108(50), 20260–20264 (2011). 22106295 10.1073/pnas.1116437108 PMC3250154

[34] Smith J, Sones K, Grace D, MacMillan S, Tarawali S, Herrero M. Beyond milk, meat, and eggs: role of livestock in food and nutrition security. Animal Front. 3(1), 6–13 (2013).

[35] The United Nations Food and Agriculture Organization (FAO). Rome Declaration on Nutrition. FAO, Rome, Italy (2014).

[36] The United Nations Food and Agriculture Organization (FAO). FAO Statistical Yearbook 2013: World Food and Agriculture. FAO, Rome, Italy (2013).

[37] The United Nations Food and Agriculture Organization (FAO). The Future of Food and Agriculture. FAO, Rome, Italy (2017).

[38] The United Nations Department of Economic and Social Affairs (UN DESA). World Population Prospects: The 2015 Revision, Key Findings and Advance Tables. United Nations, NY, USA (2015).

[39] Godfray HCJ, Beddington JR, Crute IR Food security: the challenge of feeding 9 billion people. Science 327, 812–818 (2010). 20110467 10.1126/science.1185383

[40] Van Boeckel TP, Brower C, Gilbert M Global trends in antimicrobial use in food animals. Proc. Natl Acad. Sci. USA 112(18), 5649–5654 (2015). 25792457 10.1073/pnas.1503141112 PMC4426470

[41] Bengtsson B, Greko C. Antibiotic resistance—consequences for animal health, welfare, and food production. Upsala J. Med. Sci. 119, 96–102 (2014). 24678738 10.3109/03009734.2014.901445 PMC4034566

[42] O’Neill J. Antimicrobials in agriculture and the environment: reducing unnecessary use and waste. In: Review on Antimicrobial Resistance. (2015).

[43] WHO. Antimicrobial Resistance: Global Report on Surveillance. WHO, Geneva, Switzerland (2014).

[44] Iweriebor B, Iwu C, Obi L, Nwodo U, Okoh A. Multiple antibiotic resistances among shiga toxin producing *Escherichia coli* O157 in feces of dairy cattle farms in Eastern Cape of South Africa. BMC Microbiol. 15, 213 (2015). 26475706 10.1186/s12866-015-0553-y PMC4609099

[45] Weese JS. Methicillin resistant *Staphylococcus aureus* in animals. ILAR J. 51(3), 233–244 (2010). 21131724 10.1093/ilar.51.3.233

[46] FAO. The FAO Action Plan on Antimicrobial Resistance 2016–2020. FAO, Rome, Italy (2016).

[47] Padungtod P, Kadohira M, Hill G. Livestock production and foodborne diseases from food animals in Thailand. J. Vet. Med. Sci. 70(9), 873–879 (2008). 18840959 10.1292/jvms.70.873

[48] de Balogh K, Halliday J, Lubroth J. Integrating the surveillance of animal health, foodborne pathogens and foodborne diseases in developing and in-transition countries. Rev. Sci. Tech Off. Int. Epiz. 32(2), 539–548 (2013). 10.20506/rst.32.2.2241 24547657

[49] Scharff RL. Economic burden from health losses due to foodborne illness in the United States. J. Food Prot. 75(1), 123–131 (2012). 10.4315/0362-028X.JFP-11-058 22221364

[50] Tomson G, Vlad I. The need to look at antibiotic resistance from a health systems perspective. Upsala J. Med. Sci. 119, 117–124 (2014). 24673267 10.3109/03009734.2014.902879 PMC4034548

[51] Thanner S, Drissner D, Walsh F. Antimicrobial resistance in agriculture. mBio 7(2), e02227–02215 (2016). 27094336 10.1128/mBio.02227-15 PMC4850276

[52] Berendonk TU, Manaia CM, Merlin C Tackling antibiotic resistance: the environmental framework. Nat. Rev. Microbiol. 13, 310–317 (2015). 25817583 10.1038/nrmicro3439

[53] Li B, Zhang T. Biodegradation and adsorption of antibiotics in the activated sludge process. Envion. Sci. Techno. 44(9), 3468–3473 (2010). 10.1021/es903490h 20384353

[54] Martinez JL. Environmental pollution by antibiotics and by antibiotic resistance determinants. Environ. Pollut. 157, 2893–2902 (2009). 19560847 10.1016/j.envpol.2009.05.051

[55] Jechalke S, Heuer H, Siemens J, Amelung W, Smalla K. Fate and effects of veterinary antibiotics in soil. Trends Microbiol. 22(9), 536–545 (2014). 24950802 10.1016/j.tim.2014.05.005

[56] Williams MR, Stedtfeld RD, Guo X, Hashsham SA. Antimicrobial resistance in the environment. Water Environ. Res. 88(10), 1951–1967 (2016). 27620115 10.2175/106143016X14696400495974

[57] Suzuki S, Pruden A, Virta M, Zhang T. Editorial: antibiotic resistance in aquatic systems. Front. Microbiol. 8(14), (2017). 10.3389/fmicb.2017.00014 PMC5263135 28179896

[58] RE-Act. A fact sheet from ReAct – Action on Antibiotic Resistance. (2016).

[59] Storteboom H, Arabi M, Davis JG, Crimi B, Pruden A. Tracking antibiotic resistance genes in the South Platte River basin using molecular signatures of urban, agricultural, and pristine sources. Environ. Sci. Techno. 44(19), 7397–7404 (2010). 10.1021/es101657s 20809616

[60] Zhu Y-G, Johnson TA, Su J-Q Diverse and abundant antibiotic resistance genes in chinese swine farms. Proc. Natl Acad. Sci. 110(9), 3435–3440 (2013). 23401528 10.1073/pnas.1222743110 PMC3587239

[61] Wu XL, Xiang L, Yan QY Distribution and risk assessment of quinolone antibiotics in the soils from organic vegetable farms of a subtropical city, Southern China. Sci. Total Environ. 487(1), 399–406 (2014). 24797736 10.1016/j.scitotenv.2014.04.015

[62] Huang Y, Zhang L, Tiu L, Wang HH. Characterization of antibiotic resistance in commensal bacteria from an aquaculture ecosystem. Front. Microbiol. 6, 914 (2015). 26441859 10.3389/fmicb.2015.00914 PMC4561822

[63] Azanu D, Mortey C, Darko G, Weisser JJ, Styrishave B, Abaidoo RC. Uptake of antibiotics from irrigation water by plants. Chemosphere 157, 107–114 (2016). 27213239 10.1016/j.chemosphere.2016.05.035

[64] Keating BA, Herrero M, Carberry PS, Gardner J, Cole MB. Food wedges: framing the global food demand and supply challenge towards 2050. Glob. Food Sec. 125–132 (2014).

[65] Dantas G, Sommer MO. Context matters – the complex interplay between resistome genotypes and resistance phenotypes. Curr. Opin. Microbiol. 15(5), 577–582 (2012). 22954750 10.1016/j.mib.2012.07.004

[66] Khaliq JA, Mushtaque A, Al-Wardy M, Al-Busaidi A, Choudri BS. Wastewater and sludge management and research in Oman: an overview. J. Air Waste Manag. Assoc. 67(3), 267–278 (2017). 27717294 10.1080/10962247.2016.1243595

[67] Calero-Cáceres W, Melgarejo A, Colomer-Lluch M Sludge as a potential important source of antibiotic resistance genes in both the bacterial and bacteriophage fractions. Environ. Sci. Techno. 48(13), 7602–7611 (2014). 10.1021/es501851s 24873655

[68] Xiao K-Q, Li B, Ma L Metagenomic profiles of antibiotic resistance genes in paddy soils from South China. FEMS Microbiol. Ecol. 92, fiw023 (2016). 26850156 10.1093/femsec/fiw023

[69] Udikovic-Kolic N, Wichmann F, Broderick NA, Handelsman J. Bloom of resident antibiotic-resistant bacteria in soil following manure fertilization. Proc. Natl Acad. Sci. USA 111(42), 15202–15207 (2014). 25288759 10.1073/pnas.1409836111 PMC4210343

[70] Gerber PJ, Steinfeld H, Henderson B Tackling Climate Change Through Livestock – A Global Assessment of Emissions and Mitigation Opportunities. FAO, Rome, Italy (2013).

[71] Herrero M, Henderson B, Havlik P Greenhouse gas mitigation potentials in the livestock sector. Nat. Clim. Change 6, 452–460 (2016).

[72] Schipanski ME, Bennett EM. The influence of agricultural trade and livestock production on the global phosphorus cycle. Ecosystems 15(2), 256–268 (2012).

[73] Hammer TJ, Fierer N, Hardwick B Treating cattle with antibiotics affects greenhouse gas emissions, and microbiota in dung and dung beetles. Proc. Royal Soc. B 283(1831), (2016). 10.1098/rspb.2016.0150 PMC4892788 27226475

[74] Laxminarayan R, Amábile-Cuevas CF, Cars O UN High-Level Meeting on antimicrobials—what do we need?. Lancet 388, 218–220 (2016). 27479554 10.1016/S0140-6736(16)31079-0

[75] World Health Organization (WHO). Food Safety, Fact sheet N° 399. WHO, Geneva, Switzerland (2015).

[76] United Nations. Sustainable development goals: guidelines for the Use of the SDG Logo including the Colour Wheel and 17 icons. UN, NY, USA (2020).

